# Not Charity, Nurses, but Self-Help

**Published:** 1888-08-11

**Authors:** 


### The Hospital

A Weekly Institutional Journal of

Science, Medicine, Nursing, and Philanthropy.

*For Hospitals, Asylums, Medical Practitioners, Students, Nurses, Families, Parishes, Congregations, and the Charitable Public.*

Vol. IV. No. 98.] [August 11, 1888.

### Not Charity, Nurses, but Self-Help.

After a period of abusive criticism without precedent, the imputation of motives so base as to be self-refuting, the use of assertions so frivolous as to be astounding, and an attempt to mislead by "fogging" the issue so unscrupulous as to have aroused the most burning indignation against the critics and their backers, the National Pension Fund for Nurses, at the end of the first quarter of its active existence, stands to-day upon solid ground. All that is most worthy, influential, and representative in the hospital world is to-day ranged on the side of the National Pension Fund. This is what those who have had great experience of public movements knew must be the result of a fund established, as this has been, after years of patient inquiry, and with a full and comprehensive knowledge of the facts. Indeed, we could only wish that every nurse would take an early opportunity of reading once more, in the light of what has occurred during the last few months, Mr. Burdett's first paper, read at the Society of Arts last October. Every point since raised was there anticipated, the difficulties of various kinds were fairly and fully stated, and the only possible basis upon which the National Pension Fund for Nurses could be established—*i.e.*, a provident basis, was precisely set forth. The nurses are beginning to realise this for themselves, and are coming forward in increasing numbers to invest their savings in the fund.

This is one result no doubt of the self-restraint which the Council have exhibited under great provocation, because they approached the work with malice towards none, with charity for all, with firmness in the right, as God gives them to see the right. Once more Byron's estimate of the criticism of good work by interested persons will come home to all who have followed the fierce strife of tongues which has raged round the National Pension Fund for Nurses. This is what Byron wrote in "English Bards and Scotch Reviewers," and it applies with telling aptness to those who have set themselves to prevent the nurses from helping themselves :—

> As soon
> Seek roses in December, ice in June,
> Hope constancy in wind, or corn in chaff,
> Or any other thing that's false, before
> You trust in critics.

In another part of our present issue will be found an amusing reference to the constancy of the critics, as well as proof positive that they have offered the nurses chaff for corn, but we refrain from applying the quotation further, as a lasting peace ought to be now attainable if it is not already attained.

We have said that the National Pension Fund for Nurses stands to-day upon solid ground. This is so, because nearly one half of the first thousand nurses have already arranged to take out policies, and the business to be done is so great that the Council have determined to appoint one of the ablest and most successful of insurance secretaries as manager. This gentleman, Mr. Edward Clifford, is a host in himself. His energy, his experience, his organising power, his enthusiasm and personal influence, have won for him a high position by gaining the sympathy and confidence of the most representative men in the insurance world. The first result of this appointment will be, as we announced last week, the issue of a pamphlet prospectus giving a host of information not previously available, and the immediate issue of scrip policies to those who have already decided to join the Fund. With the view of meeting the wish of many applicants, it has been determined to allow a nurse, under certain regulations, to join for sick pay only, and to accept monthly or any other payments which individual policy-holders may prefer. In due course a copy of the new prospectus will be sent to each nurse who has already applied at the office, 38, Old Jewry, E.C., for information, but anyone may obtain one on application, enclosing a stamp for postage. The success of the Fund is almost phenomenal, and it probably exceeds that of any new insurance office yet established.

So far well, but much remains to be done. We would remind nurses and their friends that Mr. Burdett explained at the outset that the National Pension Fund must not be regarded as "a question of charity, but of providence," and if nurses were not animated by a feeling "of providence and self-help, he hoped there would be no fund at all. If a great army of 20,000 working beings have not self-reliance, energy, and independence enough to build up this Fund on a financially sound basis, believing that Providence will help them, all he could say was that the time was not ripe for it." It is for the nurses to begin to secure their lasting independence by investing in the National Pension Fund for Nurses whatever they can spare, however small the sum may be (if it is only one shilling a month, providing that is in truth the utmost they can spare from their earnings at the moment), so as to show that the best of them, at any rate, are prepared to combine to provide for themselves by their own contributions and their own efforts. Thus 1*s.* 6*d.* a month paid into the Fund will provide most nurses with 10*s.* per week during illness until they are sixty years of age. We believe that if every hospital and institution nurse in the country realised this fact to-day, she would be grateful for it.

There must, however, be no mistake as to the provident basis of the National Pension Fund. None but those who join the National Pension Fund for Nurses will have any claim upon the bonus, benevolent, and other funds which are affiliated with it. The merchant princes who have already so munificently endowed the movement with £25,000 have done so on the basis of Mr. Burdett's original paper; they will only allow their money to be used to help those who manifest a proper sense of their individual responsibilities by showing a provident spirit. The circumstances of sisters and nurses, and the claims upon their slender earnings no doubt differ considerably, but after all has been said that can be said to the contrary, the fact remains that every nurse can do a little in the way of saving if she cares to do so. This little is expected of her and must be done by every person who desires to be provided for in the day of incapacity, whether from disease, accident, or old age. A new table showing what can be done by investing 1s. a month in the Fund, has been inserted in the new prospectus, to bring the Fund within the means of every nurse, however small her present earnings may be. She may not be able, at the moment, as we have pointed out already, to enter for a retiring allowance of an adequate amount payable at fifty, fifty-five, or sixty, but she can enter for something, and unless she does this she cannot complain if she finds herself friendless and unprovided for when the bad times come, as they usually do come, to the majority of the race. Saving is as much a matter of education as nursing itself, and the sooner every prudent woman enters herself as a pupil in the art, by joining the National Pension Fund the better and happier she will be. No nurse is so poor as not to be able to save something every month, and so none can truthfully say, "I cannot become a member of this great Fund."

It may be well, in conclusion, to say that we have been at some pains to ascertain, apart from the wisdom or justice of the proposition which some have put forward, whether it would be possible to establish a Nurses' Compassionate Fund, that is, a huge charitable institution granting free pensions to all nurses. Such a proposal we have always regarded as an insult to the profession, and one which no true friend of nurses would for one moment entertain. It is only necessary to point out that a sum of something like £500,000 for every one thousand nurses would have to be raised and invested to secure a small pension for each. Taking the present number of nurses at fifteen thousand—we believe there are probably twenty thousand altogether in this country at the present time—it would be necessary to raise seven millions and a half to give a pension to each of the fifteen thousand nurses from such a fund. The idea is therefore chimerical and ridiculous, and it is no doubt the knowledge that this is the case which has determined the merchant princes and many other wealthy people to make their gifts to the National Pension Fund available only for nurses who display some desire to help themselves. Every nurse may now make herself sure of a provision and a protection by joining the National Pension Fund for Nurses, if she only uses it as a savings bank, and pays in on the returnable scale but £1 a-year. Prudent women will do this without further delay.

